# Interventions to improve delivery of isoniazid preventive therapy: an overview of systematic reviews

**DOI:** 10.1186/1471-2334-14-281

**Published:** 2014-05-21

**Authors:** Lisa V Adams, Elizabeth A Talbot, Karen Odato, Heather Blunt, Karen R Steingart

**Affiliations:** 1 Infectious Disease and International Health Section, Department of Medicine, Geisel School of Medicine at Dartmouth, 1 Rope Ferry Road, Hanover, NH 03755, USA; 2 Biomedical Libraries, Geisel School of Medicine at Dartmouth, 37 Dewey Field Road, Hanover, NH 03755, USA; 3 Cochrane Infectious Diseases Group, Liverpool, UK

**Keywords:** Tuberculosis, HIV, Adherence, Latent tuberculosis infection

## Abstract

**Background:**

Uptake of isoniazid preventive therapy (IPT) to prevent tuberculosis has been poor, particularly in the highest risk populations. Interventions to improve IPT delivery could promote implementation. The large number of existing systematic reviews on treatment adherence has made drawing conclusions a challenge. To provide decision makers with the evidence they need, we performed an overview of systematic reviews to compare different organizational interventions to improve IPT delivery as measured by treatment completion among those at highest risk for the development of TB disease, namely child contacts or HIV-infected individuals.

**Methods:**

We searched the Cochrane Database of Systematic Reviews, the Database of Abstracts of Reviews of Effects (DARE), and MEDLINE up to August 15, 2012. Two authors used a standardized data extraction form and the AMSTAR instrument to independently assess each review.

**Results:**

Six reviews met inclusion criteria. Interventions included changes in the setting/site of IPT delivery, use of quality monitoring mechanisms (e.g., directly observed therapy), IPT delivery integration into other healthcare services, and use of lay health workers. Most reviews reported a combination of outcomes related to IPT adherence and treatment completion rate but without a baseline or comparison rate. Generally, we found limited evidence to demonstrate that the studied interventions improved treatment completion.

**Conclusions:**

While most of the interventions were not shown to improve IPT completion, integration of tuberculosis and HIV services yielded high treatment completion rates in some settings. The lack of data from high burden TB settings limits applicability. Further research to assess different IPT delivery interventions, including those that address barriers to care in at-risk populations, is urgently needed to identify the most effective practices for IPT delivery and TB control in high TB burden settings.

## Background

Treating latent tuberculosis infection (LTBI) with isoniazid preventive therapy (IPT) in persons who are at high risk for developing tuberculosis (TB) has been shown to decrease progression to TB disease and improve survival [1-4]. Consequently, the World Health Organization recommends a six-month course of IPT for children younger than five years who are close contacts to an adult with sputum smear-positive TB, and HIV-infected adults and children older than 12 months who are unlikely to have TB disease [5].

Despite clear international recommendations, only 8% to 20% of eligible children actually receive IPT [6]. Studies in HIV-infected populations show similarly low rates of IPT provision [7,8]. Possible reasons for this low uptake include imperfect TB/HIV care coordination for many HIV-infected IPT candidates and a past focus on TB disease diagnosis rather than prevention. There is also a lack of programmatic guidelines to identify IPT candidates, sensitive methods to exclude TB disease, and patient management tools to ensure LTBI treatment completion. Even if patients are initiated on IPT, benefit is only realized if patients complete their prescribed therapy. While barriers to IPT adherence can exist at both programmatic and patient levels, TB control programs have long recognized the need to move beyond specific patient factors and encompass broader health system changes to optimize individual patient care and outcomes.

Medication adherence is a complex human behavior, and the medical and social science literature on this topic is extensive. In such cases – where hundreds of published studies exist on an issue – systematic reviews are typically performed to formulate precise questions and to consolidate, appraise and evaluate the evidence to facilitate decision making by clinicians and policy makers. In the case of treatment adherence, several systematic reviews have examined the effectiveness of different interventions to improve adherence in various medical conditions [9-14], including tuberculosis [15,16]. Some reviews have focused on a specific intervention with patients, such as counseling and patient education [12-15], while others have looked at system changes in regimen schedules and pill packaging [11]. Several recent systematic reviews have focused on interventions specifically to improve IPT adherence, such as the use of lay health workers [9], patient education and counseling [15], and incentives and enablers [16].

Given the many strategies that could potentially improve medication adherence, determining the attributable effects and summarizing the findings of the many studies that have evaluated these different strategies would be difficult. To provide focus, in their overview of reviews, van Dulmen and colleagues have described theoretical approaches to adherence and categorized interventions on patient adherence for different medical conditions [17]. The authors define seven categories of adherence interventions: technical (e.g., changes in packaging), behavioral (e.g., use of reminders), educational (e.g., providing information to individual patients), affective (e.g., provider empathy), social support (e.g., practical or emotional support), structural (e.g., establishing a care program in the workplace) and complex or multi-faceted (e.g., some combination of the above) [17]. With treatment completion serving as our patient important outcome, we sought interventions in IPT delivery most likely to improve adherence among individual patients.

Interventions related to the delivery, practice, and organization of health care services have also been categorized by the Cochrane Effective Practice and Organisation of Care (EPOC) Group [18]. Under the EPOC taxonomy, the category “organizational interventions” are those interventions that involve “a change in who delivers health care, how care is organized, or where care is delivered” [18]. This category of interventions fits a health system’s approach to support adherence. With treatment completion as the ultimate goal of IPT delivery, organizational interventions that can be undertaken by a national TB program to affect IPT adherence may have the greatest impact. Mapping interventions to the EPOC group taxonomy ensures valid comparisons of findings are made.

With the large number of systematic reviews already performed on interventions to improve treatment adherence generally and IPT adherence specifically, the challenge persists of how to integrate, assess, compare and contrast the evidence from these reviews to facilitate clinical and programmatic decision-making. In this instance, the best approach is to summarize the reviews in one place by performing an *overview of systematic reviews*. As described by Smith et al., overviews of systematic reviews are the appropriate next step to provide clinical decision makers with the comprehensive information that they need [19].

Therefore, we conducted an overview of systematic reviews that assessed interventions to improve adherence of IPT in high-risk individuals to better inform decision making on IPT delivery. Specifically, we sought to compare different organizational interventions to improve IPT delivery as measured by treatment completion among those at highest risk for the development of TB disease, namely child contacts or HIV-infected individuals. This focused review question allowed us to concentrate on interventions that a national TB program can most easily influence. To the best of our knowledge, no such overview has been performed. These findings are intended to inform public health professionals and policy makers from high TB burden countries where IPT delivery by programs, uptake by patients, and treatment completion rates are suboptimal. Improved worldwide delivery of IPT could reduce global morbidity and mortality from TB, especially among children and HIV-infected individuals.

### Objective

Our objective was to synthesize and compare the evidence from systematic reviews examining the effect of organizational interventions on IPT delivery among those at highest risk for the development of TB disease.

## Methods

We followed the Cochrane Collaboration recommendations for performing overviews of systematic reviews as described in the Cochrane Handbook [20].

### Criteria for considering reviews for inclusion

Types of reviews: We included all systematic reviews written in English, Spanish, and French that met our selection criteria and were identified by our search strategies. We excluded narrative reviews and systematic reviews that did not include acceptable study designs (i.e., randomized controlled trials, quasi-randomized controlled trials, controlled before-and-after studies, interrupted time series or before-and-after studies); and evaluate at least one of the specified organizational interventions; or measure treatment completion.

Types of participants: Although our population of interest was child contacts and HIV-infected individuals, we included systematic reviews of all persons for whom treatment of LTBI is recommended regardless of age and HIV infection [21,22]. We broadened inclusion because we anticipated there would be limited data on child contacts and HIV-infected individuals. We included systematic reviews that focused primarily on organizations and health systems.

### Types of interventions

Interventions related to the delivery, practice, and organization of health care services have been categorized into four groups by the Cochrane Effective Practice and Organisation of Care (EPOC) Group: professional, financial, organizational, and regulatory [18]. We selected organizational interventions because these are the interventions most feasible for TB control program implementation, can be instituted without the need for considerable additional financial inputs, and are expected to be the most commonly practiced and studied interventions to improve IPT delivery. We included one provider-oriented intervention, the revision of professional roles, since this also seemed a feasible activity for a national TB program to undertake. Other types of interventions are not typically under the purview of the TB control program. The organizational, predominantly structural, interventions we considered most relevant to IPT delivery are listed in Table 1. We included reviews evaluating these interventions, or combinations of these interventions. We excluded financial and regulatory interventions.

**Table 1:** **Mapping of the included reviews to the selected cochrane effective practice and organisation of care group organizational interventions**^**a**^

**Intervention**	**Definition**	**Example of intervention**	**Reviews mapped to this category**	**Bottom-line statements of effectiveness**
Changes to the setting/site of service delivery (3 reviews)	This intervention could include providing IPT outside of the usual TB clinic setting, such as in an HIV care and treatment center for HIV-infected patients or in the household through home-based care programs for TB child contacts or in another community-based setting.	Introduction of IPT to community settings such as methadone clinics or shelters (Al-Darraji et al, 2012 [23] and DeFulio et al, 2012 [24]); integration of TB/HIV services (Uyei et al, 2011 [25])	Al-Darraji et al, 2012 [23]; DeFulio et al, 2012 [24]; Uyei et al, 2011 [25]	Overall, high levels of adherence and a range of treatment completion of IPT were reported.
Changes in medical records systems (0 reviews)	This intervention might include changing from paper to computerized records and the use of patient-tracking systems.	None	None	N/A
Presence and organization of quality monitoring mechanisms (2 reviews)	Quality monitoring mechanisms may include monitoring of medication or treatment outcomes.	Provision of directly observed therapy (DOT) for LTBI (Hirsch-Moverman et al, 2008 [26]; Zuñiga et al, 2012 [27])	Hirsch-Moverman et al, 2008 [26]; Zuñiga et al, 2012 [27]	While higher adherence was observed with DOT than self-administered therapy, actual completion rates in comparative studies remain suboptimal—as low as 44% to at best 80% (Hirsch-Moverman et al, 2008 [26]).
Revision of professional roles, e.g., nurses and lay health workers (1 review)	This intervention includes shifts in the roles among caregivers, also known as ‘professional substitution’ or shifting boundaries in professional care [28].	Use of lay health care workers for peer supported self-supervision or DOT	Lewin et al, 2010 [9]	There was moderate quality evidence that this type of support had little or no effect on the completion of IPT.
Integration of IPT delivery into primary health care services (0 review)	This intervention focuses on improving access by incorporating IPT delivery into other health care services	None	None	N/A

^**a**^These interventions are described in detail in the Cochrane Effective Practice and Organisation of Care Review Group, Data Collection Checklist, 2002, http://epoc.cochrane.org/sites/epoc.cochrane.org/files/uploads/datacollectionchecklist.pdf.

### Types of outcomes

The primary outcome of interest was treatment completion rate (TCR). We defined TCR as the percentage of patients who initiated IPT and took at least 80% of prescribed doses within a nine-month period. We also included TCR as defined by the authors of the review. IPT was defined as INH monotherapy taken at any dose for at least six months to treat LTBI.

### Search strategy

We searched the following databases without date restrictions on August 15, 2012: the Cochrane Database of Systematic Reviews (Cochrane Library 2012 issue 8), the Database of Abstracts of Reviews of Effects (DARE) (Cochrane Library 2012 issue 8) and MEDLINE (PubMed). To identify systematic reviews in MEDLINE, we used PubMed's Systematic Review filter. We searched for citations in all languages; however, due to available resources, we restricted the articles for inclusion to those published in English, French, or Spanish.

We employed two search strategies (Additional file 1): the first included indexed terms and text words related to latent tuberculosis and its drug therapies. Due to the similarities between IPT and co-trimoxazole for primary prevention of opportunistic infections in HIV-infected persons in terms of duration and target population, we also searched for systematic reviews that evaluated the delivery of co-trimoxazole preventive therapy. Therefore, the second strategy included indexed terms and text words related to HIV, pneumocystis pneumonia, and co-trimoxazole preventive therapy. Additionally, one author hand-searched reviews and overviews in four Cochrane review groups (Cochrane Infectious Diseases Group, Cochrane Effective Practice and Organisation of Care Group, Cochrane Public Health Group, Cochrane Consumers and Communication Review Group) and DARE databases through June 2012. We also checked reference lists of selected papers for additional reviews.

### Selection of reviews

Two authors independently scrutinized non-duplicate titles and abstracts identified by literature searching to identify potentially eligible reviews. Any citation identified by either author was included in a second screen during which the full text was reviewed. The same review authors independently assessed the reviews for inclusion using pre-specified inclusion and exclusion criteria. Any disagreements were resolved by discussion. One review author selected reviews identified by hand-searching the Cochrane database.

### Data collection and analysis

We developed a standardized data extraction form to summarize key characteristics of the reviews. One author piloted the form with two reviews and finalized the form. Then two authors independently extracted data on the following characteristics: objectives, study designs of included studies, participants, setting, country, interventions, outcomes, and review quality. Any disagreements in data extraction were resolved by discussion or decision of a third author. We did not contact review or study authors for additional information due to resource constraints.

### Quality assessment

We used the AMSTAR instrument to assess methodological quality of each review [29]. We did not assess the quality of the individual studies in the reviews but noted any assessment by the review authors.

### Role of the funding source

The funding source for this work did not influence the study design, interpretation of data, writing of the report, or the decision to submit the paper for publication.

## Results

**Figure 1:**
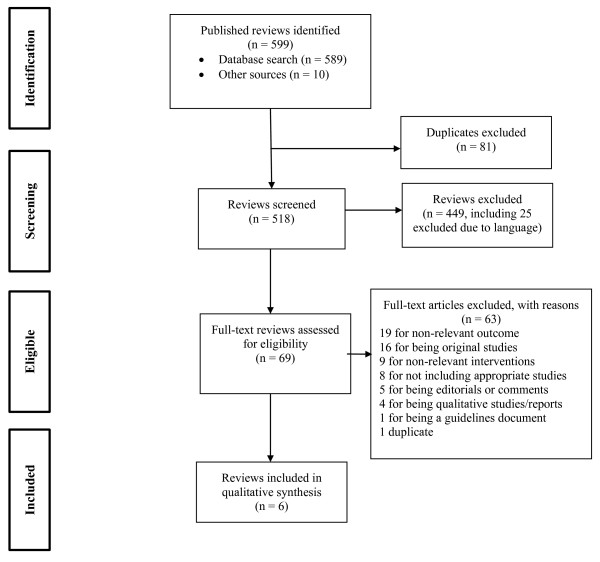
**PRISMA Flow Diagram.** Legend: The PRIMSA diagram details our search and selection process applied during the overview.

Our search yielded a total of 589 citations, with an additional nine systematic reviews identified from a review of the references of the selected reviews and one by hand-searching the Cochrane database. Figure 1 details our search and selection process. We selected reviews that, based on our inclusion criteria, included at least one organizational intervention (based on the EPOC taxonomy) to improve IPT or co-trimoxazole adherence. We identified six reviews that met our criteria. All assess IPT delivery; none of the co-trimoxazole adherence reviews identified met our intervention criteria.

Of the six selected reviews (involving a total of 105 studies), one was a Cochrane review [9] and five were non-Cochrane reviews [23-27]. The specific interventions in this overview mapped to the EPOC taxonomy are presented in Table 1. Extracted data from the six included reviews are shown in Table 2.

**Table 2:** Characteristics of included reviews

**Author, year, reference**	**Systematic review objective(s)**	**Study settings (no. of studies related to IPT delivery)**	**Participants**	**Countries where studies conducted**	**Percent studies in high TB incidence setting**^**a**^	**Outcome related to TCR**	**Applicability**
Al-Darraji, 2012 [23]	Review interventions to improve IPT delivery in correctional facilities	Jails and prisons (18)	Adults	US, Spain, Singapore	6%	Median TCR 44% (3-87%); low TCRs among RCTs (23, 12 and 12%)	Behavior of incarcerated adults may not be generalizable; incomplete HIV prevalence data
DeFulio, 2012 [24]	Review use of incentives on medication adherence	Methadone, primary care, and public clinics, homeless shelters (5)	Adults and children	US, Timor-Leste	20%	TCR improved in 2 of 3 studies (44% v. 26% and 92% v. 82%); 1 showed no effect	Behavior of addicted and homeless populations may not be generalizable; only 1 study included children, none specified inclusion of HIV populations
Hirsch-Moverman, 2008 [26]	To identify predictors of adherence and adherence interventions	Jails, refugee camp, homeless shelter, healthcare setting (19)	IV drug users, incarcerated, homeless, refugee/ foreign-born, healthcare workers, aboriginal populations	US and Canada	0%	Inconsistent across studies; no single intervention reliably showed effectiveness	Mixed adult populations; lack of regional diversity; incomplete HIV prevalence data
Lewin, 2010 [9]	To assess the effects of lay health worker interventions in primary and community health care on maternal and child health and the management of infectious diseases	US (2)	IV drug users, adolescents (ages 11-19, mostly Hispanic American)	US	0%	Little to no effect on treatment completion (RR = 1 · 0)	No children under age 5 included, 20% HIV-infected in one study, HIV status unknown in other study
Uyei, 2011 [25]	To examine the effect of strategies for TB and HIV service integration on delivery, outcomes for patients, and cost-effectiveness	Counseling and testing centers, hospitals, clinics (6)	HIV-infected patients	Botswana, South Africa, Uganda	100%	High adherence (75-92%); moderate to high TCRs (47-88%)	Unclear if children included
Zuñiga, 2012 [27]	To synthesize data on LTBI treatment adherence in Hispanic populations in the US	Clinics and public health programs dispensing LTBI treatment (12)	Hispanic adults	US	0%	Self-reports of LTBI adherence may be inaccurate; power dynamic impedes patient and healthcare provider communication; direct measures of adherence will improve validity of results	No children included; adult Hispanic populations only, no HIV data

^a^High TB incidence defined as TB incidence > 30/100,000.

IPT = isoniazid preventive therapy; TB = tuberculosis; TCR = treatment completion rate; RCT = randomized controlled trial; IV = intravenous; LTBI = latent tuberculosis infection.

The six systematic reviews assessed different interventions to improve IPT adherence and, in some cases, adherence with diagnostic testing and other treatments. Hirsch-Moverman et al. also aimed to identify predictors of adherence and Zuñiga et al. to identify correlates of IPT adherence in Hispanic patients. In assessing these two reviews, we focused on the effect of the interventions. Four of the six reviews assessed studies of IPT adherence that were conducted predominantly in the US [9,23,26,27], or the US and Canada [26], or the US, Spain (four studies) and Singapore (one study) [23], the latter being a high-income country with a TB incidence rate of 50/100,000 [30]. The most common study designs reported by the review authors were observational (30 studies), randomized controlled trials (19 studies), and prospective cohort (15 studies).

The AMSTAR ratings of the included reviews ranged from 4 to 10 out of a maximum of 11 (Table 3). Only one review was rated as high quality (AMSTAR score of 10) and three reviews were rated as lower quality (AMSTAR ratings of less than seven). Only two reviews reported on methodological quality of the included studies [9,25]. In Lewin et al., two of 82 studies addressed IPT delivery; the review authors judged that one of these studies had adequate sequence generation but was not blinded. The other study only addressed incomplete data adequately. Neither study had sufficient data for the review authors to determine the risk of bias due to allocation concealment or freedom from selective reporting. In Uyei et al., the authors’ general appraisal of the quality of studies noted the absence of randomization and that missing data were addressed in only two of the included studies.

**Table 3:** AMSTAR rating for included systematic reviews

**AMSTAR criteria**	**Use of an ’a priori’ design**	**Duplicate study selection and data extraction**	**Comprehensive searching of the literature**	**Use of publication status as an exclusion criterion**	**Provision of (included and excluded) studies**	**Provision of characteristics of included studies**	**Assessment of methodological quality of included studies**	**Appropriate use of quality of included studies in formulating conclusions**	**Appropriate methods for combining results of studies**	**Assessment of publication bias**	**Conflict of interest (both review and included studies) stated**
Al-Darraji	X	X	X	X	X	X					
DeFulio			X	X	X	X					
Hirsch-Moverman	X	X	X	X	X	X					
Lewin	X	X	X	X	X	X	X	X	X	X	
Uyei	X	X	X	X		X	X	X	X	X	
Zuñiga	X		X	X		X					

### Participants

All six reviews assessed studies that enrolled adults, and two reviews included children or adolescents (1 of 5 [20%] studies in Defulio et al. and 1 of 4 [25%] in Lewin et al.). Studies of IPT adherence in the US were focused on the populations that are at the highest risk for treatment non-adherence, namely intravenous drug users [9,23,24,26], incarcerated individuals [9,24,27], homeless individuals [23,27], and recent immigrants [24,25]. Three reviews specified that HIV-infected individuals were included in any of the included studies (9 of 18 [50%] studies in Al-Darraji et al., 9 of 78 [12%] in Hirsch-Moverman and 56 of 56 [100%] in Uyei et al.) [23,25,26].

### Interventions

The majority of interventions examined in the studies of the included systematic reviews were changes in the setting/site of service delivery. Three reviews assessed studies of integration of IPT delivery into other health care services, either community-based care [23,24] or HIV care [25]. One review assessed studies that examined the impact of a revision of professional roles, specifically the use of lay health workers to support self-administered IPT or provide directly observed therapy [9]. Two reviews assessed the impact of providing directly observed therapy [26,27], while one also looked for additional correlational relationships related to side-effects, social support, demographics, education, and self-report of health [27].

### Outcomes

Most reviews reported outcomes related to IPT adherence and TCR (Table 2). Only one review provided a clear definition of treatment completion, defining it as the ingestion of at least 80% of prescribed doses [25]. The authors of this review stated that, to allow comparisons across studies, they calculated TCR as the number completing treatment over the number initiating treatment. Another review calculated TCR as the percentage of patients who completed “six months of treatment within the study’s follow-up period” [25]. Most reviews provided only the post-intervention TCR (i.e., without the pre-intervention rate). Collectively, there was little evidence to demonstrate that most of the interventions studied had a positive impact on TCRs.

## Discussion

This overview is the first published assessment of the evidence of organizational interventions to improve IPT adherence. Among the reviews that assessed studies of IPT adherence in high-risk US populations, none of the interventions examined were found to be effective at improving IPT adherence or TCR. The systematic review by Uyei et al. that included only studies from high TB-burden, low-income settings examined the impact of TB and HIV service integration. While IPT adherence was reportedly high across the six studies in this review, the TCRs were variable. In addition, the lack of comparison groups in these studies makes it difficult to assess if TB and HIV service integration was the main driver of the benefit.

Beyond setting, the applicability of the evidence from these systematic reviews based on patient population is uncertain. While our populations of interest were child contacts and HIV-infected individuals, most reviews assessed IPT adherence in adult populations, with only two reviews including children in no more than one-quarter of their included studies [9,24]. Similarly, only three reviews included HIV-infected persons, although with greater representation among the included studies (12% - 100%) [23,25,26].

We found that the quality of the systematic reviews in this overview varied. The low quality of the majority of reviews suggests limitations in design and/or execution that may undermine conclusions about the results of the reviews.

We also recognize that there are other as yet unstudied interventions or combinations of interventions that might improve IPT adherence. In their overview of reviews of interventions focused on improving consumers’ use of non-IPT medication, Ryan et al. examined self-monitoring and self-management, simplification of dosing, and involvement of pharmacists [31]. While they found several interventions that showed promise in improving adherence, none were consistently effective across diseases, populations, or settings. It is unclear whether their findings are applicable to IPT because none of the reviews they evaluated specifically addressed IPT.

Although the contribution of educational and counseling sessions to adherence was not the primary objective of this overview, we extracted these data when available. Two reviews that met inclusion for our overview (that were not included in Ryan et al.) assessed the use of education and/or counseling as a means of improving treatment adherence in a number of settings [23,26]. Both included studies of IPT delivery but neither found a demonstrable improvement in adherence.

A recent Cochrane review by M’Imunya et al. specifically assessed the role of patient education and counseling in promoting adherence to TB treatment and LTBI treatment [15]. They conclude that the LTBI TCR may increase, but they emphasize that benefits from patient education and counseling vary with context and the underlying reasons for poor adherence. Therefore, any educational intervention must be tailored to the setting and population, and there is unlikely to be a universally beneficial approach.

We also recognize that adverse reactions and how they are managed are important to patients and can reduce adherence and treatment completion. Adverse reactions and their impact on treatment completion could not be systematically addressed in our overview as this would have required a different methodology. Nonetheless, we looked for and summarized adverse drug reactions when data were provided in the included reviews. While four of the six review authors sought to collect this data, only two reported any data on the frequency of adverse events. In both reviews, the reported rates were very low (median <5%) [23,27].

There are limitations to our overview. While the search strategy included all languages, we included only reviews published in English, French, and Spanish, and therefore may have missed papers that were published in other languages. However, of 589 citations initially identified, only 25 (4%) papers were excluded based on language. There was some overlap across the reviews that examined specific populations, namely US residents who were homeless, using intravenous drugs, and/or in prison (8 of 105 total studies [8%] appeared in more than one review). One study of Hispanic patients was included in two reviews (1/105, 1%).

## Conclusions

Overall, while most organizational interventions did not consistently result in improvements in IPT completion, the integration of TB and HIV services yielded high TCRs in some settings. The lack of data on organizational interventions to improve IPT delivery in high burden TB settings and in the high-risk populations of interest makes it difficult to draw conclusions applicable to populations in these settings. This notable absence of data is, however, a clear indication of the need for additional investigation in these settings and these populations. Further research to assess different IPT delivery interventions, including those that address barriers to care in at-risk populations, is urgently needed to identify the most effective practices for IPT delivery and TB control in high TB-burden settings.

## Abbreviations

DARE: Database of Abstracts of Reviews of Effects; EPOC: Effective Practice and Organisation of Care; IPT: Isoniazid preventive therapy; LTBI: Latent tuberculosis infection; TB: Tuberculosis; TCR: Treatment completion rate.

## Competing interests

The authors declare that they have no competing interests.

## Authors’ contributions

LVA, EAT, and KRS developed the study design. KO and HB led development of the search strategy and conducted the searches of the different databases. LVA, EAT, and KRS developed the criteria for evaluating the systematic reviews including inclusion/exclusion criteria. LVA and EAT screened the reviews identified and selected those included. LVA, EAT, and KRS interpreted the findings. All authors contributed to the writing of the manuscript. All authors read and approved the final manuscript.

## Authors’ information

KRS is an Editor with the Liverpool-based Cochrane Infectious Diseases Group, which aims to impact policy and research in tropical diseases by producing high quality, relevant, systematic reviews and to lead developments in review quality improvement and effective dissemination of findings (http://cidg.cochrane.org/).

## Pre-publication history

The pre-publication history for this paper can be accessed here:

http://www.biomedcentral.com/1471-2334/14/281/prepub

## Supplementary Material

Additional file 1 Search strategy. Click here for file
