# Supplementary material for: Interventions to improve delivery of isoniazid preventive therapy: an overview of systematic reviews
Source: BMC Infect Dis. 2014 May 21;14:281. doi: 10.1186/1471-2334-14-281 (PMC4038070; doi:10.1186/1471-2334-14-281)
Supplement: Additional file 1 — Search strategy. [file 1471-2334-14-281-S1.docx]

**Appendix 1: Search Strategy**

There were two search strategies employed in this overview. The first included indexed terms and text words related to latent tuberculosis and its drug therapies (specifically, isoniazid and rifampin). The second strategy included indexed terms and text words related to HIV, pneumocystis pneumonia, and co-trimoxazole therapy. Below are the terms that were used for each search.

**Latent TB/Isoniazid/Rifampin strategy**

**MEDLINE (PubMed)**

Search "Isoniazid"[Mesh] OR "Rifampin"[Mesh] OR "Latent Tuberculosis"[Mesh] OR IPT[tiab] OR Isoniazid[tiab] OR INH[tiab] OR "Isonicotinic Acid Hydrazide"[tiab] OR Phthivazide[tiab] OR Ftivazide[tiab] OR Tubazide[tiab] OR Isonex[tiab] OR Rifampin[tiab] OR Rifampicin[tiab] OR Benemycin[tiab] OR Tubocin[tiab] OR Rimactane[tiab] OR Rifadin[tiab] OR Rimactan[tiab] OR Rifapentine[tiab] OR LTBI[tiab] OR "latent tuberculosis"[tiab] OR "latent TB"[tiab] OR "latent multiple-drug resistant tuberculosis"[tiab] AND Systematic[sb]

**Cochrane Database of Systematic Reviews and Database of Abstracts of Reviews of Effects (DARE)**

#1 MeSH descriptor: [Isoniazid] this term only

#2 MeSH descriptor: [Rifampin] this term only

#3 MeSH descriptor: [Latent Tuberculosis] this term only

#4 (IPT or Isoniazid or INH or "Isonicotinic Acid Hydrazide" or Phthivazide or Ftivazide or Tubazide or Isonex or Rifampin or Rifampicin or Benemycin or Tubocin or Rimactane or Rifadin or Rimactan or Rifapentine or LTBI or "latent tuberculosis" or "latent TB" or "latent multiple-drug resistant tuberculosis"):ti,ab,kw

#5 #1 OR #2 OR #3 OR #4

in Cochrane Reviews (Reviews and Protocols) and Other Reviews

**HIV/Pneumocystis Pneumonia/Co-trimoxazole Therapy strategy**

**MEDLINE (PubMed)**

Search (HIV[mesh] OR "HIV Infections"[mesh] OR HIV[tiab] OR "human immunodeficiency virus"[tiab] OR "human immunodeficiency viruses"[tiab] OR AIDS[tiab] OR "acquired immunodeficiency syndrome"[tiab] OR "acquired immune deficiency syndrome"[tiab])

AND

(Trimethoprim-Sulfamethoxazole combination[mesh] OR Pneumonia, pneumocystis[mesh] OR "trimethoprin-sulfa"[tiab] OR "trimethoprim-sulfamethoxazole"[tiab] OR CPT[tiab] OR "co-trim"[tiab] OR cotrim[tiab] OR Centran[tiab] OR Centrin[tiab] OR "Co-Trimoxazole"[tiab] OR "Co Trimoxazole"[tiab] OR Cotrimoxazole[tiab] OR Duclor[tiab] OR "Trimethoprim Sulfamethoxazole"[tiab] OR Insozalin[tiab] OR "Sulfamethoxazole-Trimethoprim"[tiab] OR "Sulfamethoxazole Trimethoprim"[tiab] OR "TMP SMX"[tiab] OR "TMP-SMX"[tiab] OR Trimedin[tiab] OR Trimezole[tiab] OR Eslectin[tiab] OR Bactifor[tiab] OR Bactrim[tiab] OR Biseptol[tiab] OR "Biseptol-480"[tiab] OR "Biseptol 480"[tiab] OR "Biseptol480"[tiab] OR Drylin[tiab] OR Eusaprim[tiab] OR Kepinol[tiab] OR Lescot[tiab] OR Metomide[tiab] OR Oriprim[tiab] OR Septra[tiab] OR Septrin[tiab] OR Sulfatrim[tiab] OR Sulprim[tiab] OR Sultrex[tiab] OR Sumetrolim[tiab] OR Trimethoprimsulfa[tiab] OR Trimeth-sulfa[tiab] OR Trimosulfa[tiab] OR Abactrim[tiab] OR Uroplus[tiab] OR PCP[tiab] OR Pneumocystis[tiab] OR jirovecii[tiab] OR jiroveci[tiab] OR carinii[tiab] OR pneumocystosis[tiab] OR pneumocystoses[tiab]) AND Systematic[sb]

**Cochrane Database of Systematic Reviews and Database of Abstracts of Reviews of Effects (DARE)**

#1 MeSH descriptor: [HIV] explode all trees

#2 MeSH descriptor: [HIV Infections] explode all trees

#3 HIV or "human immunodeficiency virus" or "human immunodeficiency viruses" or AIDS or "acquired immunodeficiency syndrome" or "acquired immune deficiency syndrome":ti,ab,kw

#4 #1 OR #2 OR #3

#5 MeSH descriptor: [Trimethoprim-Sulfamethoxazole Combination] explode all trees

#6 MeSH descriptor: [Pneumonia, Pneumocystis] explode all trees

#7 "trimethoprin-sulfa" or "trimethoprim-sulfamethoxazole" or CPT or "co-trim" or cotrim or Centran or Centrin or "Co-Trimoxazole" or "Co Trimoxazole" or Cotrimoxazole or Duclor or "Trimethoprim Sulfamethoxazole" or Insozalin or "Sulfamethoxazole-Trimethoprim" or "Sulfamethoxazole Trimethoprim" or "TMP SMX" or "TMP-SMX" or Trimedin or Trimezole or Eslectin or Bactifor or Bactrim or Biseptol or "Biseptol-480" or "Biseptol 480" or "Biseptol480" or Drylin or Eusaprim or Kepinol or Lescot or Metomide or Oriprim or Septra or Septrin or Sulfatrim or Sulprim or Sultrex or Sumetrolim or Trimethoprimsulfa or Trimeth-sulfa or Trimosulfa or Abactrim or Uroplus or PCP or Pneumocystis or jirovecii or jiroveci or carinii or pneumocystosis or pneumocystoses:ti,ab,kw

#8 #5 OR #6 OR #7

#9 #8 AND #4

in Cochrane Reviews (Reviews and Protocols) and Other Reviews
